# A comprehensive assessment of cerebellar damage in multiple sclerosis using diffusion tractography and volumetric analysis

**DOI:** 10.1177/1352458511403528

**Published:** 2011-09

**Authors:** VM Anderson, CAM Wheeler-Kingshott, K Abdel-Aziz, DH Miller, A Toosy, AJ Thompson, O Ciccarelli

**Affiliations:** 1 Department of Neuroinflammation, UCL Institute of Neurology, London, UK.; 2 Department of Brain Repair and Rehabilitation, UCL Institute of Neurology, London, UK.

**Keywords:** atrophy, cerebellum, chronic progressive multiple sclerosis, diffusion tensor imaging, magnetic resonance imaging, relapsing–remitting multiple sclerosis

## Abstract

**Background:** White matter (WM) and grey matter (GM) brain damage in multiple sclerosis (MS) is widespread, but the extent of cerebellar involvement and impact on disability needs to be clarified.

**Objective:** This study aimed to assess cerebellar WM and GM atrophy and the degree of fibre coherence in the main cerebellar connections, and their contribution to disability in relapsing–remitting MS (RRMS) and primary progressive MS (PPMS).

**Methods:** Fourteen patients with RRMS, 12 patients with PPMS and 16 healthy controls were recruited. Cerebellar WM and GM volumes and tractography-derived measures from the middle and superior cerebellar peduncles, including fractional anisotropy (FA), mean diffusivity (MD), and directional diffusivities, were quantified from magnetic resonance imaging (MRI). Patients were assessed on clinical scores, including the MS Functional Composite score subtests. Linear regression models were used to compare imaging measures between 12 RRMS, 11 PPMS and 16 controls, and investigate their association with clinical scores.

**Results:** Patients with PPMS showed reduced FA and increased radial diffusivity in the middle cerebellar peduncle compared with controls and patients with RRMS. In PPMS, lower cerebellar WM volume was associated with worse performance on the upper limb test. In the same patient group, we found significant relationships between superior cerebellar peduncle FA and upper limb function, and between superior cerebellar peduncle FA, MD and radial diffusivity and speed of walking.

**Conclusion:** These findings indicate reduced fibre coherence in the main cerebellar connections, and link damage in the whole cerebellar WM, and, in particular, in the superior cerebellar peduncle, to motor deficit in PPMS.

## Introduction

Multiple sclerosis (MS) is a disabling neurological disease, characterized by inflammatory demyelinating lesions within the white matter (WM) of the brain and spinal cord, but also involving demyelination and neuroaxonal loss within the grey matter (GM) and ‘normal-appearing’ WM.^1^^–^^4^ Magnetic resonance imaging (MRI) has enabled investigation into the extent of diffuse pathological damage in patients with MS using a number of techniques including high-resolution structural sequences, from which WM and GM volumes can be calculated, and diffusion tensor imaging (DTI), from which parameters sensitive to the integrity of WM fibres are derived, such as fractional anisotropy (FA). Reduced volume (or atrophy) in patients compared with controls is thought to reflect neuroaxonal loss,^4,5^ whilst reduced FA is thought to represent axonal loss and demyelination.^6,7^ FA information can be measured along specific WM tracts by means of diffusion-based tractography, which uses the information contained in the DTI to track entire pathways in vivo.^8^

The cerebellum plays a major role in motor function, and tissue damage within the cerebellum is thought to contribute to disability in patients with MS. Cerebellar atrophy is more extensive in patients with secondary progressive MS and those with longer disease duration when compared with people who have relapsing–remitting (RR) MS and/or shorter disease duration, and cerebellar atrophy has been shown to correlate with clinical measures of disability.^9,10^ The extent of involvement of the cerebellar cortex specifically has been of particular interest in recent years. Post-mortem studies on MS tissue samples have shown demyelination in the cerebellar cortex,^11,12^ whilst significant atrophy of the cerebellar cortex measured from MRI in patients with both relapsing and progressive MS has been reported.^13,14^ However, whether there is clinically relevant atrophy in the cerebellar cortex of patients with primary progressive (PP) MS needs further investigation.

With respect to cerebellar WM, post-mortem studies have shown demyelination in patients with MS,^11,12^ but to a lesser extent than the demyelination observed in cerebellar GM. Whilst previous MRI studies have found reductions in the volume of cerebellar WM in MS patients compared with controls,^13,15^ there is no information about the extent of WM atrophy in PPMS. Extensive WM changes, characterized by reduced FA, have been observed across the brains of patients with PPMS and RRMS using tract-based spatial statistics, which compares groups on a voxel-wise basis.^16,17^ However, whether WM damage can be detected in the main cerebellar connections in vivo in patients with MS has not been investigated.

From a pathophysiological perspective, it is important to determine whether there is an association between pathological damage in the WM and that in the GM, or whether the two are affected independently. Some studies have previously shown an association between pathological processes occurring in the WM and GM in some regions of the cerebral hemispheres in patients with PPMS.^16,18^ However, no correlation between demyelination in the cerebellar cortex and that in the WM was found in a post-mortem study,^12^ suggesting that the mechanisms of damage in the WM and GM may change across the brain regions or across MS subtypes.

Thus, the aims of this study were to use MRI to assess the differences in WM and GM volumes and tractography-derived measures in the main cerebellar connections between patients with RRMS and PPMS and healthy controls, and to investigate the relative contribution of cerebellar WM and GM injury in determining disability in both groups of patients. The associations between WM and GM damage in both groups of patients are also explored. The middle and superior cerebellar peduncles were chosen for tractography reconstruction and DTI-indices measurement because: (i) the middle cerebellar peduncle (MCP) is the largest afferent system of the cerebellum with fibres ending in the cerebellar cortex (i.e. cortico-ponto-cerebellar pathway); (ii) the superior cerebellar peduncle (SCP) contains afferent tracts to the cerebellum and efferent fibres from the cerebellar nuclei, which influence parts of the brain stem and the thalamus; (iii) both peduncles are well defined anatomically, and are clinically relevant to motor function.^19,20^

## Methods

### Subjects and clinical assessment

In total, 26 patients with MS (14 RRMS, mean age 44.9 years (SD 11.9), seven males; 12 PPMS, mean age 55.6 years (SD 9.8), six males) and 16 healthy control subjects (mean age 40.4 years (SD 13.2), seven males) were included in the study and underwent the same MRI protocol. Healthy controls were age-matched to the RRMS patients, but not the PPMS patients; therefore, age was included in each step of the statistical analysis (see below). One patient with PPMS felt unwell during the scanning and did not complete the protocol; two patients with RRMS were later excluded (see below), leaving a total of 23 patients (Table 1). All patients were clinically assessed on the day of MR scanning using the Expanded Disability Status Scale (EDSS),^21^ which included the cerebellar functional systems score (C-FSS). Clinical assessment also included the Timed 25-foot Walk Test (TWT)^22^ and the Nine-Hole Peg Test (9-HPT).^23^ The average of two trials of the TWT, and the average of four trials of the 9-HPT (averaged as reciprocals of the mean times from two trials for each hand) were calculated.^24^

**Table 1.** Subject characteristics of the patients who contributed to the final results

	RRMS (*n* = 12)	PPMS (*n* = 11)
Age, years (mean (SD))	45.0 (12.9)	56.3 (10.0)
Gender	7 males, 5 females	5 males, 6 females
Disease duration, years (mean (SD))	8.8 (7.8)	12.6 (7.6)
EDSS (median (range))	4.5 (2.0–6.5)	5.0 (2.5–7.5)
Cerebellar-FSS (median (range))	0 (0–2)	2 (0–4)
25-foot timed walk, s (mean/median (range))	8.3 / 8.4 (4.9–12.0)	26.2 / 7.9 (4.8–180)
Nine-hole peg test, s (mean/median (range))	36.5 / 22.7 (19.6–158.6)	38.3 / 23.2 (18.9–162.7)

Cerebellar-FSS = Cerebellar Functional System Score.

Ethical approval was granted by the Joint Medical Ethics Committee of the National Hospital for Neurology and Neurosurgery and the Institute of Neurology, London. All subjects gave informed, written consent.

### MR image acquisition and analysis

MR imaging was performed on a 1.5 T Signa Echospeed scanner (GE Medical Systems, Milwaukee, WI, USA). All subjects had brain T1-weighted inversion-recovery prepared fast spoiled-gradient recall (FSPGR) imaging (TR 14.3 ms, TE 5.1 ms, TI 450 ms, flip angle 20°, matrix size 256 × 128, field of view 310 × 160 mm^2^, 160 contiguous coronal slices, voxel resolution 1.2 × 1.2 × 1.2 mm). DTI data were acquired using a single-shot diffusion-weighted (DW) echo planar imaging sequence (FOV 220 × 220 mm, matrix 96 × 96 reconstructed to 128 × 128, in-plane resolution 2.3 × 2.3 mm^2^ reconstructed to 1.7 × 1.7 mm^2^, 60 contiguous axial slices, 2.3 mm slice thickness, cardiac gating (TR 20RR (≈20 s)), diffusion gradients applied along 61 optimized directions with a maximum *b* factor of 1200 s/mm^2^, 7 *b *≈ 0 s/mm^2^ images).^25,26^

T1-weighted volumetric MRI were segmented into GM, WM and cerebrospinal fluid (CSF) using SPM5 (Statistical Parametric Mapping, Wellcome Trust Centre for Neuroimaging, Institute of Neurology, http://www.fil.ion.ucl.ac.uk/spm).^27^ Binary GM and WM masks were converted into regions-of-interest which were overlaid on the original MRI, and the cerebellar tissue, corrected for lesion mis-classification, was delineated on coronal slices as described previously.^13^ In addition, total intracranial volume (TIV) was calculated for each subject as the sum of the GM, WM, CSF and lesion volumes obtained from SPM processing.

**Figure 1.**
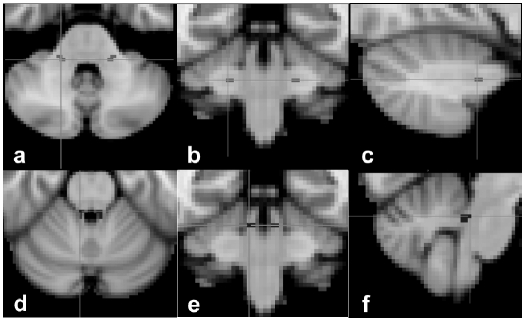
The seed masks of the middle cerebellar peduncle (a)–(c) and superior cerebellar peduncle (d)–(f) overlaid onto the MNI-152 T1-weighted brain image, in axial view (a) and (d), coronal view (b) and (e), and sagittal view (c) and (f).

The processing of the diffusion data and the tractography analysis were performed using tools available in the FSL Library (http://www.fmrib.ox.ac.uk/fsl). The diffusion data were processed using FDT (FMRIB’s Diffusion Toolbox) v2.0.^28^ Following data conversion, DW images were corrected for motion and eddy current distortion. A diffusion tensor model was fitted on a voxel-by-voxel basis and local modelling of the diffusion parameters was performed using Markov chain Monte Carlo sampling to build up a probability distribution function of the primary diffusion direction at each voxel.^28^ The probabilistic tractography algorithm provided by FSL^28^ was used to reconstruct the MCP and SCP. The MCP represents the largest of the three cerebellar peduncles, and contains afferent axons that originate from the pontine nuclei, contributing to the cortico–ponto–cerebellar pathway. The MCP mediates complex brain functions, as it allows a flow of information from the cerebral cortex to the cerebellum.^29^ The SCP contains not only afferent fibres to the cerebellum (i.e. ventral spinocerebellar tract), but also efferent fibres from the dentate nucleus to the brain stem and thalamus (i.e. the dentatorubral and dentatothalamic tracts), which ultimately act on the lower motor neurons.^30^ For the MCP, two seed masks were positioned over the middle part of the left and right MCP on the standard MNI-152 T1-weighted (2 mm) brain image (MNI coordinates for left MCP *x* = 53, *y* = 45, *z* = 18; for right MCP *x* = 36, *y* = 45, *z* = 18), while for the SCP, two seed masks were designed over the middle part of the left and right SCP on the same template (MNI coordinates for left SCP *x* = 48, *y* = 44, *z* = 23; for right SCP *x* = 41, *y* = 44, *z* = 23) (Figure 1).

**Figure 2.**
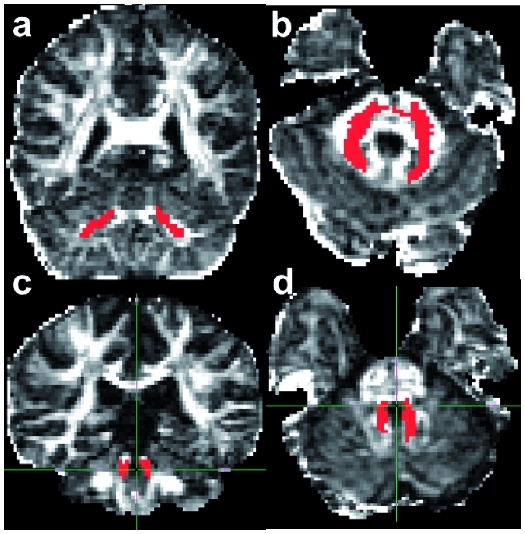
Example of the tractography reconstruction of the middle cerebellar peduncle (a) and (b) and superior cerebellar peduncle (c) and (d) overlaid on the fractional anisotrophy image in coronal view (a) and (c) and axial view (b) and (d).

These seed masks were then transformed into the individual space of DW images using FLIRT. The transformation matrix was based on the inverse and concatenated transformation matrices obtained from linear registration of the non-DW image to the T1-weighted FSPGR image and the linear registration of the T1-weighted FSPGR image to the MNI-152 brain image. This automated approach was taken to avoid introducing bias that could have resulted from manually placing seed masks on each subject’s image independently, and was thus 100% reproducible. The correct location of seed masks in native space was confirmed by visual inspection in all cases. Probabilistic tractography was used to reconstruct fibres passing through the seed masks. Exclusion masks were placed in the sagittal midline, at the inferior limit of the temporal lobes, and at the anterior and superior limits of the cerebellum, to constrain the tractography algorithm. To exclude voxels with low connectivity, which may have resulted from image noise and therefore not reflect the underlying structure, the resulting connectivity maps of the MCP and those of the SCP were thresholded to include voxels with connectivity values of 200 and 100, respectively, on a scale from 0–20,000, as thresholding has largely been used in brain studies.^31^^–^^33^ The SCP was characterized by a lower connectivity than the MCP, and therefore a lower threshold (100) was used to obtain a plausible tract, as seen in Figure 2.

From all voxels within the seed mask, the tractography algorithm generated 5000 individual pathways based on the probability distributions on principle fibre direction, with a curvature threshold of 0.2 (corresponding to a minimum angle of approximately ± 80°). Finally, the probability of a course within a given voxel was represented by the sum of these samples passing through it. For each tract, the trajectory obtained was checked to ensure the location was anatomically plausible (Figure 2); the orientation of the principal eigenvector in the tract was also visually checked in all subjects to ensure that they were consistent with the known anatomy and across subjects.^34^ In two patients with RRMS, the connectivity of one of the reconstructed tracts was too low to survive thresholding, and these data were therefore excluded from the statistical analysis. One of these two patients showed a lesion in the pons and a lesion in the cerebral peduncle on the side where tract connectivity did not survive thresholding, despite showing an MCP which appeared to be properly reconstructed; therefore, we cannot exclude that these lesions contributed to reduce the value of connectivity below the chosen threshold. The average connectivity, mean diffusivity (MD), fractional anisotropy (FA), axial diffusivity (λ1) and radial diffusivity (average of λ2 and λ3) within the left and right MCP and SCP were calculated.

### Statistical analysis

All data were analysed using STATA version 9 (StataCorp, College Station, TX, USA, http://www.stata.com). We found no statistically significant differences in tractography-derived measurements of both cerebellar peduncles between the left and right sides, and therefore measurements were averaged for each peduncle, in each subject. Linear regression models were used to compare cerebellar volumes and tractography-derived measures of both the middle and superior cerebellar peduncles between subject groups, using a group indicator, and controlling for age and gender by including them as covariates. TIV was also entered as a covariate when investigating differences in cerebellar volumes in order to normalize for differences in head size, whilst the number of voxels in the tract was entered as a covariate when investigating differences in tract-derived measures.

The relative contribution of MRI measures of WM damage (DTI-derived measures and normalized cerebellar WM volume) and GM damage (normalized cerebellar GM volume) to disability, as measured by the 9-HPT and TWT, was investigated in the MS patients using linear regression analysis, with the inverse of the scores used (i.e. a more positive score represented less disability), to make the scores normally distributed. The association of age and gender with test scores was determined in univariate analyses, and they were entered into the regression analyses as covariates if the association was significant; the number of tract voxels was also entered when investigating the contribution of DTI-derived measures of both cerebellar peduncles to test scores. Ordinal logistic regression was used to assess the relative contributions of GM and WM damage to C-FSS and EDSS score. To assess whether tractography-derived values of the MCP and SCP (MD, FA, axial and radial diffusivity) were associated with normalized cerebellar GM volume in the patient groups, linear regression was used to model the data, with age, gender and number of voxels added as covariates to control for the effect of these factors.

## Results

### Differences in cerebellar imaging measures between groups

Mean normalized cerebellar GM and WM volumes and tractography-derived measures in the MCP and SCP are shown in Table 2. Two small lesions adjacent to the 4th ventricle on the left side were seen in two patients, and two bilateral lesions in the same location were seen in another patient; however, in all these cases the tractography-derived MCP and SCP appeared to be correctly reconstructed.

**Table 2.** Mean corrected values (adjusted for age, gender, total intracranial volume/number of tract voxels, as explained in the text) and (SD) of cerebellar grey matter and white matter volumes and diffusion-tractography measures in the middle and superior cerebellar peduncles in controls, patients with relapsing remitting multiple sclerosis (RRMS), and patients with primary progressive multiple sclerosis (PPMS)

	Controls (*n* = 16)	RRMS (*n* = 12)	PPMS (*n* = 11)
Cerebellar GM volume (cm^3^)	97.5 (9.2)	97.2 (7.6)	93.3 (16.2)
Cerebellar WM volume (cm^3^)	20.4 (1.7)	20.8 (3.2)	18.9 (3.4)
**Middle cerebellar peduncle**			
Fractional anisotropy	0.88 (0.12)	0.86 (0.17)	0.79 (0.13)
Mean diffusivity (mm^2^/s × 10^−3^)	1.20 (0.066)	1.23 (0.080)	1.24 (0.090)
Radial diffusivity (mm^2^/s × 10^−3^)	0.89 (0.086)	0.93 (0.13)	0.97 (0.12)
Axial diffusivity (mm^2^/s × 10^−3^)	1.82 (0.11)	1.84 (0.071)	1.79 (0.095)
**Superior cerebellar peduncle**			
Fractional anisotropy	0.43 (0.03)	0.42 (0.04)	0.41 (0.05)
Mean diffusivity (mm^2^/s × 10^−3^)	0.97 (0.14)	1.54 (0.11)	1.03 (0.10)
Radial diffusivity (mm^2^/s × 10^−3^)	0.64 (0.12)	0.69 (0.09)	0.70 (0.09)
Axial diffusivity (mm^2^/s × 10^−3^)	1.25 (0.19)	1.36 (0.15)	1.32 (0.15)

GM, grey matter; WM, white matter.

Patients with PPMS showed a statistically significant decrease in MCP FA (mean difference −0.09 (−10.7%) 95% Confidence Intervals (CI) −0.15, −0.02, *p* = 0.012) and a significant increase in MCP radial diffusivity (mean difference 0.726 × 10^−5^ (7.7%), 95% CI 0.97 × 10^−5^, 0.13 × 10^−3^, *p* = 0.025) when compared with controls, after correcting for age, gender, and number of MCP voxels (Table 2). MCP FA was also significantly lower in patients with PPMS compared with patients with RRMS (mean difference −0.08 (95% CI −0.15, −0.005, *p* = 0.037). There were no statistically significant differences in any other diffusion measures of the MCP or SCP, or in WM or GM cerebellar volumes between patients with PPMS and controls. There were no significant differences in any of the MRI measures between patients with RRMS and controls.

### Contribution of cerebellar GM and WM injury to disability

**Figure 3.**
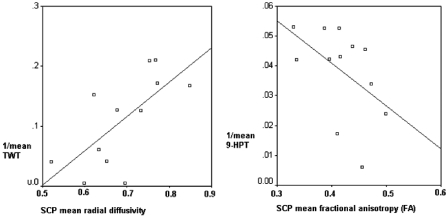
Correlation between diffusion parameters of the superior cerebellar peduncle (SCP) and motor scores, as assessed by the inverse of TWT and 9-HPT, in patients with primary progressive multiple sclerosis. 9-HPT, Nine-Hole Peg Test; FA, fractional anisotropy; SCP, superior cerebellar peduncle; TWT, Timed 25-foot Walk Test.

Cerebellar WM volume was associated with 9-HPT score in patients with PPMS, independently of cerebellar GM volume. For each 10 mm^3^ decrease in cerebellar WM volume, the time taken to complete the 9-HPT increased by 3.1 s (95% CI 1.6, 40.8; *p* = 0.038). In the same group of patients, the following correlations between tractography-derived measures of the SCP and clinical scores, including TWT and 9-HPT, were found, when adjusting for age, gender and number of voxels in the SCP: (i) FA was associated with the inverse of TWT (beta −0.0012, 95% CI −0.002, 0.0001, *p* = 0.047); (ii) MD was associated with the inverse of TWT (beta 0.40, 95% CI 0.035, 0.78, *p* = 0.035); (iii) RD was associated with the inverse of TWT (beta 0.5, 95% CI 0.12, 0.88, *p* = 0.016) (Figure 3); (iv) FA was associated with the inverse of 9-HPT (beta −0.15, 95% CI −0.28, −0.011, *p* = 0.037) (Figure 3). No other associations were observed between the tractography-derived measures of the MCP or SCP, or cerebellar GM volume, and performance on the clinical tests. In particular, no association was found between MRI measures of cerebellar damage and C-FSS.

### Association between tissue damage in the cerebellar GM and WM

Correcting for age, gender and the number of tract voxels, there was no significant association of normalized cerebellar GM volumes with tractography-derived values of the MCP and SCP in patients with either RRMS or PPMS.

## Discussion

We have performed a comprehensive assessment of cerebellar involvement in MS using diffusion tractography and volumetric analysis, and investigated its impact on disability. Our main findings were: (i) patients with PPMS showed reduced FA, which was mainly driven by increased radial diffusivity, in the MCP when compared with healthy controls and patients with RRMS; (ii) cerebellar WM volume was associated with worse performance on the upper limb motor task in patients with PPMS; (iii) in patients with PPMS, there were significant associations between SCP FA and upper limb function, and between SCP FA and MD and walking ability. This latter relationship was mainly driven by SCP radial diffusivity. We also explored the association between WM and GM damage in the cerebellum, and found that the extent of damage in the WM was independent from that in the GM in both groups of patients. We will discuss these findings in turn.

Our finding of reduced FA in the MCP in patients with PPMS extends the results of previous studies, which have reported a widespread decrease in FA across the brain,^35,36^ by showing that the main cerebellar connections, which contribute to associative, limbic, sensory and motor circuits, are affected.^29^ Therefore, diffusion-based tractography is able to reflect underlying pathological changes and may complement the information obtained with voxel-based analyses.^16^ In addition, our analysis suggests that the detected decrease in FA was due to an increase in radial diffusivity which, despite reflecting the water diffusion perpendicular to the main direction of fibres, and therefore being a marker of demyelination in animal studies,^37^ is not considered to selectively measure demyelination in patient studies.^38^

Surprisingly, we did not detect significant differences in any MRI-derived measure between patients with RRMS and controls. This could indicate that pathological changes in the cerebellar tracts in this group are subtle and below the sensitivity of the technique, or that the relatively small sample size may have limited the detection of group differences (as suggested by the wide 95% CIs obtained), as previous studies have shown both reductions in GM in patients with RRMS (which correlated with reduced performance on the 9-HPT) and decreases in FA in infratentorial normal-appearing WM.^13,39^ Alternatively, it could be that, in this particular RRMS cohort, there were genuinely no differences in cerebellar characteristics when compared with controls, and that the involvement of other motor-related pathways would therefore account for the impaired motor disability scores. Focal inflammatory demyelination in WM appears to predominate in people with RRMS, whilst more diffuse WM pathology is evidenced in PPMS,^1^ and our results in subjects with RRMS may therefore reflect a lack of diffuse changes within normal-appearing WM.

In patients with PPMS, we found that decreased cerebellar WM volume was associated with reduced performance on the 9-HPT independently of cerebellar GM volume. However, since this group of patients showed a lower cerebellar WM volume when compared with controls which did not reach statistical significance, this result must be taken with caution and needs to be confirmed by further study. Although we have previously shown an association of cerebellar WM volume with performance on the 9-HPT in patients with RRMS and secondary progressive MS, our earlier study found that this was not independent of cerebellar GM volume, which is in contrast to the current study.^13^ Interestingly, the same association with 9-HPT was seen when the diffusion parameters of the SCP were used, in particular FA. This upper limb test requires manual dexterity, and therefore it is likely that WM injury leads to a slowing of motor function. In addition, FA, MD and, above all, radial diffusivity of the SCP, significantly correlated with speed of walking, as measured by the TWT. This suggests that not only damage to the whole cerebellar WM, but also that of the SCP, play a role in determining disability. The C-FSS, which is part of the EDSS, did not show any association with imaging measures of the cerebellar peduncles, which may be in agreement with the reports that interruption, even surgical transection, of either spinocerebellar or cerebro-ponto-cerebellar pathways causes little, or only transient, cerebellar dysfunction.^30^ On the other hand, interruption of dentatothalamic and dentatorubral tracts (which run in the SCP) may cause an intentional tremor^30^ that affects the upper limb function (which is well captured by the 9-HPT).

With regard to investigating the association between GM and WM damage, we did not find any significant relationship between MCP and SCP diffusion parameters and cerebellar GM volume, suggesting that the observed WM damage in the cerebellum is (at least in part) independent from GM damage. This finding supports the hypothesis that the relationship between injury in these two compartments may vary across brain regions; for example, it may be correlated when the motor pathway and the associated motor cortex are taken into account,^16^ but is independent when the cerebellar peduncles and the associated cerebellar cortex are considered, as seen in this study. However, our results have to be taken cautiously and considered to be preliminary, as in our investigation no statistically significant differences in WM and GM volumes were observed in people with MS compared with controls. A key question which will be addressed in further studies is whether cerebellar GM lesions are associated with cerebellar WM damage, and whether imaging parameters including diffusion, measured in the cerebellar and hemispheric cortices, which receive the afferent fibres passing through the cerebellar peduncles, are associated with damage in these fibres.

An important methodological consideration is that a large number of statistical tests (about 95) were performed without formal correction for statistical comparisons. However, since we tested the hypotheses that imaging measures in the main cerebellar connections differ between patients and controls, and that cerebellar WM and GM injury contributes to disability, this study can be considered to be hypotheses-driven rather than truly exploratory; for this type of study, the need for multiple comparisons correction is less relevant.^40^ Nevertheless, we realise that it is important for future studies to confirm the result reported here in order to increase our confidence that cerebellar WM injury is important in determining disability in patients with PPMS. In addition, from a methodological point of view, recent advances in tractography algorithms and analysis, the introduction of non-linear registration techniques, and the increased use of 3 T clinical scanners, may allow one to use tractography to test new hypotheses and provide novel insights into the organization of the whole cortico–ponto–cerebellar system and the effects on it induced by brain diseases.^41^

In conclusion, these findings extend previous studies in PPMS by showing that reduced fibre coherence affects the main cerebellar connections, which mediate important brain functions, and that the whole cerebellar WM volume, which potentially reflects axonal loss, and the damage in the SCP fibres, may play a role in determining motor dysfunction, as assessed by the upper limb function test and speed of walking. In addition, in both groups of patients, injury in the GM and WM appears to occur independently in the cerebellum, suggesting that the association between GM and WM pathology may vary with brain region.
